# Electricity in Post Partum Hemorrhage

**Published:** 1888-02

**Authors:** 


**Electricity in Post Partum Hemorrhage.**—The hemorrhage was controlled, after other means failed, by the application of the current to the womb ; one pole being held by the patient, while the other was held by the physician, who grasped the womb with the opposite hand.

